# Non-Vesicular Release of Alarmin Prothymosin α Complex Associated with Annexin-2 Flop-Out

**DOI:** 10.3390/cells12121569

**Published:** 2023-06-06

**Authors:** Hiroshi Ueda

**Affiliations:** 1 Department and Institute of Pharmacology, National Defense Medical Center, Nei-hu, Taipei 114201, Taiwan; ueda1hiroshi@icloud.com; 2 Department of Pharmacology and Therapeutic Innovation, Graduate School of Biomedical Sciences, Nagasaki University, Nagasaki 852-8521, Japan

**Keywords:** DAMPs, alarmins, GSDMD, MLKL, exosomes, SNARE complex, S100A13, scramblase, flippase

## Abstract

Nuclear protein prothymosin α (ProTα) is a unique member of damage-associated molecular patterns (DAMPs)/alarmins. ProTα prevents neuronal necrosis by causing a cell death mode switch in serum-starving or ischemic/reperfusion models in vitro and in vivo. Underlying receptor mechanisms include Toll-like receptor 4 (TLR4) and G_i_-coupled receptor. Recent studies have revealed that the mode of the fatal stress-induced extracellular release of nuclear ProTα from cortical neurons in primary cultures, astrocytes and C6 glioma cells has two steps: ATP loss-induced nuclear release and the Ca^2+^-mediated formation of a multiple protein complex and its extracellular release. Under the serum-starving condition, ProTα is diffused from the nucleus throughout the cell due to the ATP loss-induced impairment of importin α–mediated nuclear transport. Subsequent mechanisms are all Ca^2+^-dependent. They include the formation of a protein complex with ProTα, S100A13, p40 Syt-1 and Annexin A2 (ANXA2); the fusion of the protein complex to the plasma membrane via p40 Syt-1–Stx-1 interaction; and TMEM16F scramblase-mediated ANXA2 flop-out. Subsequently, the protein complex is extracellularly released, leaving ANXA2 on the outer cell surface. The ANXA2 is then flipped in by a force of ATP8A2 activity, and the non-vesicular release of protein complex is repeated. Thus, the ANXA2 flop-out could play key roles in a new type of non-vesicular and non-classical release for DAMPs/alarmins, which is distinct from the modes conducted via gasdermin D or mixed-lineage kinase domain-like pseudokinase pores.

## 1. Introduction

Damage-associated molecular patterns (DAMPs)/alarmins are extracellularly released from the cell upon various types of stress and exert inflammatory or inflammation-related actions. Representative DAMPs/alarmins include high mobility group box 1 (HMGB1) protein, heat shock proteins, extracellular cold-inducible RNA-binding protein (eCIRP) and various isoforms of S100 proteins [1,2,3,4]. As most of them are known to have important physiological roles in the cell [5,6,7,8], the mechanisms underlying the extracellular release of DAMPs/alarmins have attracted the concerns of many investigators. Of interest is the fact that most of these proteins are released in a way of non-classical and non-vesicular release, which is distinct from exocytosis, as seen in neurotransmitters and peptide hormones. Current studies have revealed that some of representative DAMPs/alarmins use the release modes via gasdermin D (GSDMD) and mixed-lineage kinase domain-like pseudokinase (MLKL) pores [9,10]. In the present review, the author introduces a new mechanism, the annexin A2 (ANXA2) flop-out mediated release of prothymosin α, a neuroprotective member of DAMPs/alarmins [11], as well as GSDMD or MLKL pore-mediated non-classical and non-vesicular release.

## 2. Various Modes of Extracellular Release

### 2.1. Classical Release Modes for Biologically Active Molecules

The classical mode of secretion mechanisms has been discussed as exocytosis. It is a regulated type of exocytosis of hormones, neurotransmitters and digestive enzymes. In neurons and endocrine cells, a small percentage of secretory vesicles are fused with the plasma membrane upon cell stimuli, whereas the majority remains in a filamentous network of synapsins or actin in the case of neurons and endocrine cells, respectively, in reserve for subsequent stimulation [12]. In these mechanisms, several Ca^2+^-binding proteins are involved in molecular processes, such as tethering, docking, priming and fusion, in which Ca^2+^ sensor synaptotagmin-1, vesicular SNARE synaptobrevin (and homologue) and target SNAREs syntaxin/SNAP-25 (and homologues) play key roles in vesicle docking and fusion to the plasma membrane of nerve endings. Details have been reported elsewhere [13].

### 2.2. Non-Classical Constitutive Vesicular Release

Unlike regulated exocytosis, there is a non-classical or constitutive vesicular exocytosis during the secretion of materials, such as collagen and extracellular matrix proteins (fibroblasts) [14] or bone matrix proteins (osteoblasts) [15]. Calcium signaling and cytoskeletal dynamics are also involved via a variety of signaling pathways and cellular processes. There are two constitutive types of vesicular release: exosome release and lysosome-mediated release. They are generated via a fusion of multivesicular bodies (MVBs) with plasma membranes and released into the extracellular space [16]. Exosomes play roles as carriers of several molecules, such as DNA, RNAs (miRNAs and small RNAs), proteins (signaling proteins and heat shock proteins) and lipids. Some of them are incorporated into other cells and exert biological actions [16]. The biogenesis of exosomes begins with the budding of endosomes, which is followed by the formation of MVBs and either the extracellular release of exosomes or their degradation at lysosomes. The manner of extracellular release uses the process of vesicular docking and fusion with SNAREs complexes and the endosomal sorting complex required for transport (ESCRT), consisting of ESCRT-0, I, II and III and the ATPase Vps4 complex [17]. On the other hand, lysosomes are also part of extracellular release. Digested and waste materials in lysosomes are extracellularly released into and contained in the extracellular matrix or are fused with the membrane.

### 2.3. Non-Classical and Non-Vesicular Release Mediated via GSDMD and MLKL Pore Formation

Bacterial endotoxin lipopolysaccharides (LPS), when administered to the body at high levels, cause septic shock. LPS is known to stimulate Toll-like receptor 4 and produce proinflammatory cytokines such as IL-1β and TNFα via the activation of NF-κB. LPS is also reported to produce IL-1β and IL-18 via the activation of caspase-1 and release these cytokines through a GSDMD pore in the plasma membrane [18]. LPS-mediated cytokine (IL-1β or IL-18) release is mediated through a membrane pore made of GSDMD N-terminus peptide oligomers, which are cleaved by activated caspase-1. The GSDMD pore also causes a membrane rupture and pyroptotic cell death, which further enhances cytokine release. However, the GSDMD pore does not allow the release of the high mobility group protein B1 (HMGB-1) caspase-1 p20 subunit due to its pore size limit [19]. On the other hand, HMGB-1 is released from necrotic, necroptotic, pyroptotic, and ferroptotic cells [20,21,22]. Necroptosis occurs in various diseases including tumor necrosis factor (TNF)-mediated systemic inflammation and ischemic reperfusion injury. Activated TNF receptor-1 (TNFR-1) triggers signaling complex 1, composed of TNFR associated via the death domain (TRADD), TNFR-associated factor 2 (TRAF2), receptor-interacting protein kinase 1 (RIPK1), cellular inhibitors of apoptosis (cIAPs), the linear ubiquitin chain assembly complex (LUBAC), transforming growth factor-β-activated kinase 1 (TAK1), and the inhibitor of κB kinase (IKK) complex, which activate NF-κB and mitogen-activated protein kinase (MAPK) [23,24]. When NF-κB activation is blocked, TRADD is dissociated from complex I, forms complex II and activates caspase 8, which promotes apoptosis. When caspase 8 activity is further blocked, complex II evolves into a necrosome, composed of RIPK1, RIPK3 and MLKL, leading to the phosphorylation of MLKL and the subsequent formation of the membrane pore made of MLKL oligomers. These mechanisms cause a necroptosis and membrane rupture for the release of larger DAMPs/alarmins, such as HMGB-1 [24]. Regarding HMGB-1 release, it is also reported that all-thiol type HMGB-1 is released from the ruptured membrane, while disulfide type HMGB-1 is released by lysosome-mediated exocytosis [25].

## 3. New Type of Non-Classical and Non-Vesicular Release

### 3.1. Identification of Prothymosin α Causing Cell Death Mode Switch

**Figure 1:**
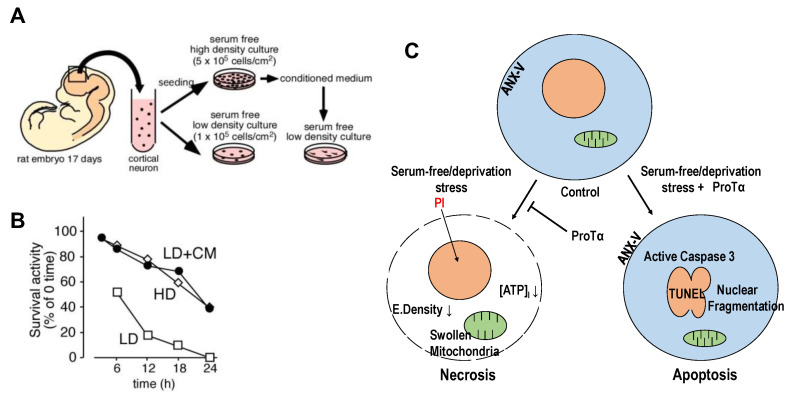
Prothymosin α as endogenous necrosis inhibitor released from neurons in starving condition. (**A**). Preparation of conditioned medium (CM) from the primary culture of embryonic (E17) rat cortical neurons at 5 × 10^5^ cells/cm^2^ (HD) cultured in the absence of serum. (**B**). Increase in survival activity of neurons cultured at a low-density (LD, 1 × 10^5^ cells/cm^2^) by the addition of CM from HD culture [26]. (**C**). Schematic changes in cell death mode of cortical neurons by recombinant ProTα. In the absence of serum, freshly prepared cortical neurons showed features of necrosis, characterized by a decrease in electron density and ATP levels, in the cytosol and swollen mitochondria and by propidium iodide (PI) incorporation into the nucleus through the disrupted plasma membrane. The addition of ProTα converted the cell death mode into apoptosis at the time point of 12 h, which is characterized by nuclear fragmentation, annexin-V (ANX-V) flop-out and caspase 3 activation.

We discovered prothymosin α (ProTα), which inhibits necrotic neuronal death, from a conditioned medium of cortical neurons [26] and observed that it is released from neurons and astrocytes upon starving or ischemia–reperfusion stress in a unique non-classical and non-vesicular manner [27]. When freshly prepared cortical neurons from 17-day-old embryonic rat brains were cultured in serum-free (no supplement) and low-density (1 × 10^5^ cells/cm^2^, LD) conditions (Figure 1A), more than 80% of neurons died in a manner of necrosis within 12 h (Figure 1B). Transmission electron microscopy (TEM) showed that there is a decrease in the electrical density of cytosol, a damaged plasma membrane and swollen mitochondria, while substantially no change in the nucleus, all of which indicate necrosis. The nature of neuronal death was also characterized by cytochemical analyses using propidium iodide (PI, necrosis marker). Of interest is the finding that neurons cultured in 5 × 10^5^ cells/cm^2^ (HD) showed an increase in survival activity; apoptotic features, such as nuclear fragmentation (TEM); and immunocytochemical changes, including the externalization of annexin V and activated caspase-3 [26]. When the conditioned medium (CM) from neurons in the HD culture was added to the LD culture, the survival activity was markedly increased (Figure 1B), and the PI signal was largely inhibited. Using a cytochemical assay with PI staining for the screening, we purified the necrosis-inhibitory factor from the CM and identified that it was nuclear protein ProTα [26]. The addition of recombinant ProTα reproduced not only the inhibition of necrotic features but also caused apoptotic features (Figure 1C). It should be noted that the apoptosis caused by ProTα is prevented by the addition of growth factors, such as brain-derived neurotrophic factor (BDNF), as shown in an in vitro culture study using cortical neurons [26] and an in vivo study in a rat middle cerebral artery occlusion (MCAO) model [28] and a mouse retinal ischemia–reperfusion model, respectively [29].

### 3.2. Serum-Free Starvation-Induced Extracellular Release of ProTα

**Figure 2:**
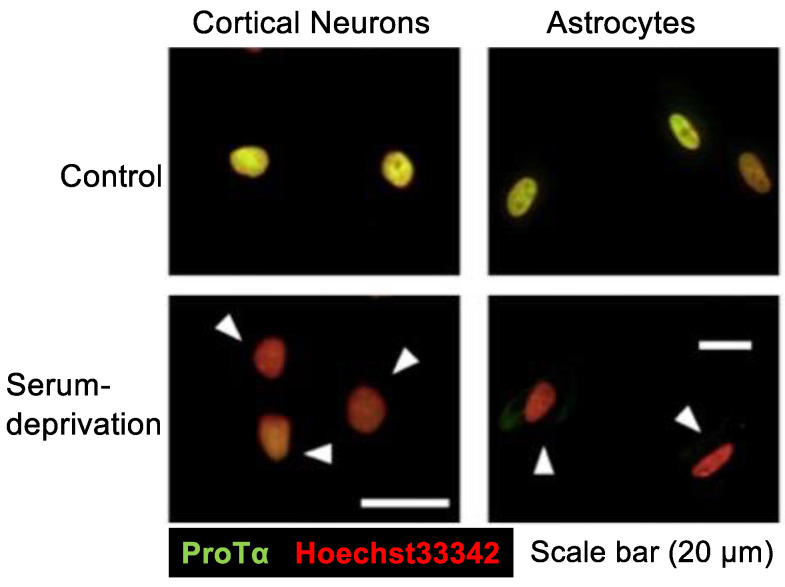
Serum deprivation-induced loss of ProTα in the nuclei of neurons and astrocytes. Results show that serum deprivation stress caused a loss of ProTα in the nuclei of cortical neurons and astrocytes in primary culture, while no significant ProTα immunoreactivity was observed in the cytosol. Details are described in a previous report [27].

In the serum-free culture of primary neurons, ProTα release into the CM started as early as 1 h after the start of culturing, and it was time-dependent till 12 h [26]. Immunocytochemical and Western blot analyses revealed that the level of ProTα in the nuclei of neurons and astrocytes was markedly decreased (Figure 2). At 3 h, cell contents were reduced to a little less than 50% of the initial cell contents of cortical neurons and astrocytes, while CM levels were a little more than 50% of the initial cell contents [27]. However, in any condition, ProTα was not detected in the cytosol (Figure 2). As no PI signals in the nucleus were observed in serum-free cultured neurons at 3 h [26], ProTα release is unlikely caused by a plasma membrane rupture. As released ProTα is considered to have beneficial survival effects in the brain [30], it is interesting to examine the mode of ProTα release. As ProTα levels in the CM from the culture of neurons and astrocytes were not affected by the pretreatment with brefeldin A, which inhibits protein transport from the endoplasmic reticulum to the Golgi complex [31], it is presumed that the mode of ProTα release is not vesicular. For the purpose of detailed cellular and molecular mechanisms of ProTα release, we used C6 glioma cells to study the serum deprivation-induced extracellular ProTα release since the release was not also affected by brefeldin A [27].

#### 3.2.1. Ischemic ATP Loss-Induced ProTα Release from the Nucleus

The mechanisms of non-classical and non-vesicular ProTα release induced by serum deprivation in C6 glioma cells could be divided into two stages. The first stage is related to the loss of cellular ATP under the condition of serum deprivation [27]. The addition of 2-deoxy-D-glucose (2-DG) reproduced ATP loss just like in the case of serum deprivation and caused a redistribution of ProTα throughout the cell by inhibiting nuclear localization. However, the extracellular release of ProTα was not observed. The intracellular injection of anti-importin α neutralizing IgG also caused a loss of nuclear localization of ProTα. This finding may be explained by the report that the loss of cellular ATP causes an inhibition of the continuous transport of NLS-possessing proteins into the nucleus [32]. It should be noted that anti-importin α IgG redistributed ProTα throughout the cell and inhibited extracellular ProTα release. The mechanisms underlying ProTα redistribution remain elusive, but it may be attributed to a passive diffusion from the nucleus since the intranuclear injection of wheat germ agglutinin, an inhibitor of the nuclear pore complex, also inhibited the nuclear localization of ProTα and caused redistribution throughout the cell [27]. Regarding the loss of the extracellular release of ProTα, additional mechanisms may be involved, as described below.

#### 3.2.2. Ca^2+^-Dependent ProTα–S100A13 Interaction

A pull-down assay using CM from serum-deprived C6 glioma cells and anti-ProTα IgG detected two protein bands at 14 and 10 kDa using Coomassie Brilliant Blue staining. The 14 kD protein was identified as ProTα by immunoblotting with anti-ProTα IgG, while the protein at 10 kDa was identified as S100A13 by MALDI-TOF MS/MS analysis [27]. In the immunocytochemistry, S100A13 was found throughout the C6 cells under the normal condition, but ProTα was localized in the nucleus. However, both proteins were completely lost from the cell following serum deprivation. Interestingly, the loss of both proteins was reversed by the addition of amlexanox (Amx), an inhibitor of S100A13 [33]; however, ProTα still remained throughout the cell, supporting the view that ProTα release is not performed in a passive fashion via membrane rupture. The quartz crystal microbalance (QCM) assay showed that ProTα and S100A13, a Ca^2+^-binding protein, interact at a ratio of 1:1; the interaction was Ca^2+^ concentration-dependent and in the range of 0.1 to 200 μM. The K_D_ value for *Strep*-tagII-S100A13 in the binding to GST-ProTα was 211.9 and 69.8 nM in the absence and presence of 100 μM Ca^2+^, respectively. In the study using deletion mutant proteins, C-terminal regions of ProTα (102–111) and S100A13 (88–98) were found to play the most important roles in the interaction. In the fluorescence resonance energy transfer (FRET) analysis using ProTα-EGFP and DsRed2-S100A13, the FRET signal was markedly increased following serum deprivation stress in the presence of amlexanox, which inhibits the release of both proteins [27]. As expected, the FRET signal was reversed by BAPTA-AM, which chelates the intracellular free Ca^2+^. It should be noted that the decrease in extracellular Ca^2+^ levels by the addition of EGTA also reversed the FRET increase, which is consistent with the previous finding that serum deprivation causes a Ca^2+^ influx via voltage-dependent N-type Ca^2+^ channels [34]. These results suggest that the next step after the nuclear release of ProTα would be related to the increase in the intracellular Ca^2+^-concentration, possibly due to the serum-deprivation-stress-induced Ca^2+^ influx via the voltage-dependent N-type Ca^2+^ channel. When C6 glioma cells expressing S100A13 (Δ88–98) were used, serum deprivation did not show the extracellular release of ProTα, and the S100A13 mutant was lost [27]. This finding suggests that serum-deprivation-stress-induced ProTα release requires the interaction with S100A13 but that S100A13 release does not require the interaction. In other words, S100A13 could be a ‘cargo’ protein for the serum-deprivation-induced release. The non-classical and non-vesicular corelease of S100A13 was also observed in the cases of fibroblast growth factor (FGF-1) and interleukin-1α [34,35,36,37,38,39]. These findings suggest that S100A13 may be a cargo protein for non-classical and non-vesicular release.

#### 3.2.3. Anti-Apoptosis Action of Cytosol ProTα

ProTα is in the nuclei of neurons, astrocytes and C6 glioma cells in culture in the absence of serum-free or serum deprivation stress, which causes necrosis and causes the extracellular release of ProTα. However, when C6 glioma cells were given apoptosis-inducing chemicals, such as staurosporin, tunicamycin or etoposide, ProTα was no more localized in the nucleus but distributed throughout the cell [27]. These changes were reversed by zDEVD-fmk, a caspase-3 inhibitor. Furthermore, unlike in the case of serum deprivation, there was no significant ProTα release in the CM. Western blot and MALDI-TOF analyses revealed that ProTα loses its C-terminal peptide (102–112), which includes NLS, a caspase-3 cleavage site and the peptide sequence required for binding to S100A13 [27]. Thus, the distinct localization of ProTα upon the apoptotic stimuli is closely related to the C-terminal specific cleavage of ProTα by caspase-3. Interestingly, one study reported that ProTα inhibits apoptosis by binding to Apaf-1, a protein component of apoptosome in non-neuronal HeLa cells [40]. Therefore, ProTα that lacks C-terminal regions may inhibit apoptosis by binding to Apaf-1 and inhibiting the formation of apoptosomes.

#### 3.2.4. Ca^2+^-Dependent Interaction between S100A13 and p40 Syt-1

To examine the molecules that interact with S100A13 in the presence of Ca^2+^, we attempted to perform pull-down experiments using StrepTactin^TM^ MicroPrep^®^ resin beads and cytosol fraction from C6 glioma cells expressing *Strep*-tagII-S100A13. As S100A13 plays a role as a cargo molecule for the non-classical release of many proteins, it was expected that many molecules would be obtained in the pull-down assay. For this reason, we followed a previous study [39], in which S100A13 interacts with fibroblast growth factor-1 (FGF-1) and Ca^2+^ sensor synaptotagmin-1 (Syt-1). Syt-1 is known to play in the facilitation of SNARE complex formation [41]. In the immunoblot experiment using materials pulled down from the C6 glioma cell cytosol fraction and anti-Syt-1 IgG in a Ca^2+^-dependent manner, a single positive band was detected at 40 kDa, corresponding to p40 Syt-1, which is deficient of the membrane-spanning domain of p65 Syt-1 [42]. When C6 glioma cells were treated with serum deprivation stress, the cell contents of ProTα and S100A13 decreased, while p65 and p40 Syt-1 levels were completely lost. On the other hand, ProTα, S100A13 and p40 Syt-1 were detected in the CM, while p65 Syt-1 was not. The detailed mechanisms remain elusive, but p65 Syt-1 may be degraded or cleaved into p40 Syt-1 by serum deprivation stress. In the surface plasmon resonance (SPR) analysis using His_6_-p40 Syt-1 attached to an anti-His_6_ IgG-coated sensor chip, *Strep*-tagII-S100A13 interacts with His_6_-p40 Syt-1 in a Ca^2+^ concentration-dependent manner with EC50 (Ca^2+^) = 83.19 μM. Under the condition of 100 μM Ca^2+^, the K_D_ of S100A13 vs. His_6_-p40 Syt-1 was 17.5 μM, which is higher than in the case of the QCM assay, in which the K_D_ of *Strep*-tagII-S100A13 vs. GST-ProTα was 69.8 nM in the presence of 100 μM Ca^2+^ [27]. Considering the involvement of N-type voltage-dependent Ca^2+^ channel and the Ca^2+^-induced Ca^2+^ release from an endoplasmic or sarcoplasmic reticulum [43], it is plausible that the interaction between S100A13 and Syt-1 may occur in the vicinity of plasma membranes, where higher Ca^2+^ concentrations are developed under the condition of serum deprivation stress. Compared with this, the interaction between ProTα and S100A13 may occur by the time of the development of high levels of Ca^2+^.

#### 3.2.5. Syt-1 Involved in the Release of ProTα and S100A13

We used the Duolink™ in situ proximity ligation assay (in situ PLA, Olink Bioscience, Uppsala, Sweden) for the detection, visualization, and quantification of the S100A13 and Syt-1 interaction. In this assay, both proteins in the fixed cell preparation were first labeled with specially designed oligonucleotides conjugated with each antibody/IgG against S100A13 and Syt-1. The preparation was then used for the amplification of the signal by generating a DNA surrogate of the protein using a polymerase. For the purpose of detecting the serum-deprivation-induced intracellular interaction of both proteins, amlexanox is useful to prevent the extracellular release. In the presence of amlexanox, the S100A13–Syt-1 interaction became evident throughout the cell time-dependently at 1.5 and 3 h after the start of serum deprivation. Furthermore, the intracellular injection of Syt-1 IgG also prevented the stress-induced release of ProTα and S100A13 [42]. All these findings suggest that Syt-1 plays a key role in the serum-deprivation-induced extracellular release of ProTα and S100A13.

#### 3.2.6. Interaction between p40 Syt-1 and Target SNARE Syntaxin-1

Syt-1 (p65 form) is a Ca^2+^ sensor [44] and promotes the formation of the SNARE complex or SNAREpin [45], which is composed of synaptobrevin, a vesicular-soluble N-ethylmaleimide-sensitive factor attachment protein receptor (v-SNARE), and target-SNARE (t-SNARE) syntaxin-1 (and its homologues), which is in the plasma membrane. The v-SNARE plays roles in the translocation and fusion of vesicles to target plasma membranes via an interaction with t-SNAREs, which include syntaxin-1 (Stx-1), SNAP-25 and SNAP-23. During neurotransmitter release via exocytosis, Syt-1 first increases the vesicle docking rate by binding to the t-SNARE/phosphatidylinositol 4,5-bisphosphate complex. Subsequently v-SNARE displaces Syt-1 from the so-called “SNAREpin”, a complex between the vesicular membrane and the target plasma membrane [46], under the low concentration of Ca^2+^. When neurons are activated and Ca^2+^ influx occurs, Syt-1 rebinds to the SNAREpin via voltage-gated Ca^2+^ channels. Thus, these Ca^2+^-dependent processes may trigger synchronous membrane fusion [47].

Based on the comparison to exocytotic vesicular release, we speculated the possible binding of p40 Syt-1 to t-SNAREs in the serum-deprivation-induced release of ProTα and S100A13 by the use of different species of Clostridium botulinum neurotoxins (BoNTs), such as type A, B and C1 [48]. Among them, the pretreatment (3 h) with BoNT/C1, which cleaves Syntaxin-1 (Stx-1), inhibited the release of ProTα, S100A13 and Syt-1, while ProTα showed a distribution throughout the cell. The specificity was observed in the finding that large amounts of S100A13 and Syt-1 were lost due to serum deprivation in C6 glioma cells pretreated with heat-inactivated BoNT1. The intracellular delivery of anti-Stx-1 IgG also blocked the release of ProTα, S100A13 and Syt-1. In addition, the treatment with siRNA for Stx-1 also blocked ProTα release [42]. The fact that substantially all Syt-1 immunoreactivities were lost due to serum deprivation may suggest the possibility that p65 Syt-1 is digested in a soluble form of p40 Syt-1, which, in turn, is released. More precise studies about the fate of p65 Syt-1 should be conducted in the future.

#### 3.2.7. p40-Syt-1-Stimulation of S100A13 Interaction with Annexin A2

Annexins play roles in Ca^2+^-mediated cellular processes, such as exocytosis-related membrane traffic, endocytosis and mitotic signaling [49,50]. Annexin A2 (ANXA2), which is one member of this family that is structurally related to actin- and phospholipid-binding proteins, is known to be an essential partner of exocytosis in neuroendocrine cells [51,52] and a functional link to the SNAREpin via S100A10, a member of divergent member of the S100 protein family [53]. When we attempted to perform the pull-down assay using C6 glioma cell lysates and *Strep*-tagII-S100A13, a member of the S100A family in the absence of Ca^2+^, ANXA2 dimer was detected, while in the presence of 100 μM Ca^2+^, higher amounts of ANXA2 dimer and β-actin were detected. On the other hand, an immunoblot assay revealed that serum deprivation caused a time-dependent (0.5–3 h) decrease in S100A13 cell contents but an increase in CM contents. However, there was no change in ANXA2 cell contents, suggesting that S100A13 is extracellularly released, while ANXA2 remains in the cell. In the ELISA-based protein binding assay, the interaction between His_6_-ANXA2 and *Strep*-tagII-S100A13 bound to the Streptavidin plate was increased in a His_6_-p40 Syt-1 concentration-dependent manner in the presence of Ca^2+^.

#### 3.2.8. Annexin A2 Flop-Out

Under serum deprivation stress, S100A13 is extracellularly released from C6 glioma cells, but ANXA2 remains in the cell in the immunoblot analysis; however, there is a tight interaction between both proteins in the cytosol of C6 glioma cells in the presence of p40 Syt-1 and Ca^2+^ in the ELISA-based protein-binding assay [11]. The tight interaction between both proteins was supported by the in situ PLA assay, in which intense amplified signals were observed 1.5 and 3 h after the serum deprivation of C6 glioma cells in the presence of amlexanox, which prevents the extracellular release of S100A13. During immunofluorescent microscopy, permeabilized preparations showed both S100A13 and ANXA2 in the cell in the presence of serum, but only ANXA2 was detected in serum-deprived preparations. On the other hand, the preparations without permeabilization did not show signals of either protein in the presence of serum, but only ANXA2 was detected in serum-deprived preparations. All these findings suggest that ANXA2, which is capable of binding to acidic phosphatidylserine (PS), is localized at the outer surface of the cell (flop-out) due to serum deprivation stress, while S100A13 is extracellularly released. Furthermore, the study using EGTA and ω-conotoxin revealed that the power of ANXA2 flop-out as well as ProTα release is mediated by the extracellular Ca^2+^ influx via the N-type voltage-dependent Ca^2+^ channel. The involvement of ANXA2 flop-out in the serum-deprivation-induced release of ProTα, S100A13 and p40 Syt-1 was evidenced by the study using the intracellular delivery of anti-ANXA2 IgG. Interestingly, both S100A13 and Syt-1 signals were localized at the edge of the plasma membrane even in the absence of amlexanox. Similarly, ProTα was detected throughout the cell but was not localized in the nucleus.

#### 3.2.9. Possible Machineries of ANXA2 Flop-Out

The mechanisms underlying ANXA2 flop-out are interesting topics to investigate. One of the mechanisms would be related to the Ca^2+^-dependent scrambling of acidic phospholipid PS in the membrane. As the asymmetrical localization of phosphatidylserine (PS) is maintained by P4-type ATPases (flippases) [54], the ATP loss due to serum deprivation stress may lead to a move-back of PS to the outer leaflet of the plasma membrane. On the other hand, we observed that serum deprivation or ischemia–reperfusion stress also causes Ca^2+^ influx in C6 glioma cells [34], which may lead to an activation of phospholipid scramblase TMEM16F [55,56]. Furthermore, there are interesting reports that ANXA2 is translocated across membranes via plasma membrane phospholipid (PS) remodeling [57] and that PS membrane domain clustering is induced by ANXA2/S100A10 heterotetramer [58]. When C6 glioma cells were pretreated with various types of P4-ATPase siRNA, ANXA2 flop-out was significantly abolished only by ATP8A2 siRNA [11]. The treatment with siRNA for ATP8A2 as well as Stx-1A and ANXA2 also significantly decreased oxygen glucose deprivation (ODG)-type ischemia and reperfusion-induced ProTα release [11]. These findings suggest that once externalized PS-ANXA2 (flop-out) would be flipped in by ATP8A2 and used for the subsequent and repeated extracellular release of the S100A13 complex in C6 glioma cells. This may explain why serum deprivation or ischemia–reperfusion induces a complete depletion of ProTα and S100A13, unlike in the case of exocytotic neurotransmitter release.

**Figure 3:**
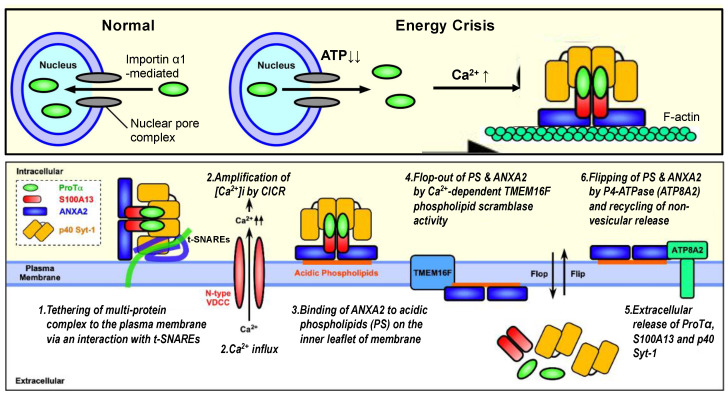
Schematic illustration of stress-induced extracellular release of ProTα, S100A13 and p40 Syt-1 (working hypothesis). *Upper panel*: Under the normal condition, importin α transports ProTα possessing nuclear localization sequence into the nucleus. The loss of ATP by starving stress impairs the importin α action for the nuclear transport of ProTα, and existing ProTα in the nucleus is then diffused throughout the cell. The starving stress also causes Ca^2+^ influx, which triggers the formation of protein complex comprising ProTα, S100A13, Syt-1 and ANXA2 on filamentous F-actin network. *Lower panel*: The protein complex is tethered to the plasma membrane with the help of interaction between p40 Syt-1 and Stx-1 (*stage 1*). High levels of intracellular Ca^2+^ caused by CICR (*stage 2*) facilitate the tight binding of the protein complex to plasma membrane with the help of interaction between ANXA2 and acidic phospholipids (e.g., phosphatidylserine/PS) (*stage 3*), followed by the TMEM16F-mediated flop-out of ANXA2-PS complex (*stage 4*) and the extracellular release of protein complex (*stage 5*). Externalized ANXA2-PS will be flipped in by a force of ATP8A2, and the additional non-vesicular release of protein complex will be repeated (*stage 6*). Details are described in the text and have been reported previously [11].

From these findings, the author proposes a new type of non-vesicular release mechanism for ProTα, a member of DAMPs/alarmins upon fatal stress. According to this working hypothesis, the initial step is the formation of the S100A13 complex (with ProTα, p40 Syt-1 and ANXA2), as shown in Figure 3, *upper panel*, as follows: (1) under the serum-deprivation condition in culture or ischemia–reperfusion stress in vivo, ProTα is diffused from the nucleus throughout the cell due to an ischemic ATP decrease (energy crisis); (2) at the same time, a Ca^2+^ influx occurs via N-type voltage-dependent Ca^2+^ channels; (3) S100A13 is then associated with cytosol ProTα, Syt-1 (p65 or p40) and ANXA2 in a Ca^2+^-dependent manner. The second step is the membrane association and extracellular release of the ProTα/S100A13 complex (Figure 3, *lower panel*), as follows: (1) the tethering of the S100A13 complex to the membrane with the help of the Ca^2+^-dependent association between Ca^2+^ sensor Syt-1 and t-SNARE Stx-1; (2) the amplification of the intracellular concentration of Ca^2+^ via a Ca^2+^-induced Ca^2+^ release (CICR) from the endoplasmic reticulum; (3) the tight binding of ANXA2 to PS on the inner leaflet of the membrane; (4) the PS-ANXA2 flop-out by Ca^2+^-dependent TMEM16F scramblase activity; (5) the extracellular release of ProTα, S100A13 and p40Syt-1 complexes; (6) the recycling of PS-ANXA2 by flipping in via a force of ATP8A2. This mechanism may be involved in the repeated ProTα/S100A13 complex release.

#### 3.2.10. Cell-Type-Specific Non-Classical and Non-Vesicular Release

**Figure 4:**
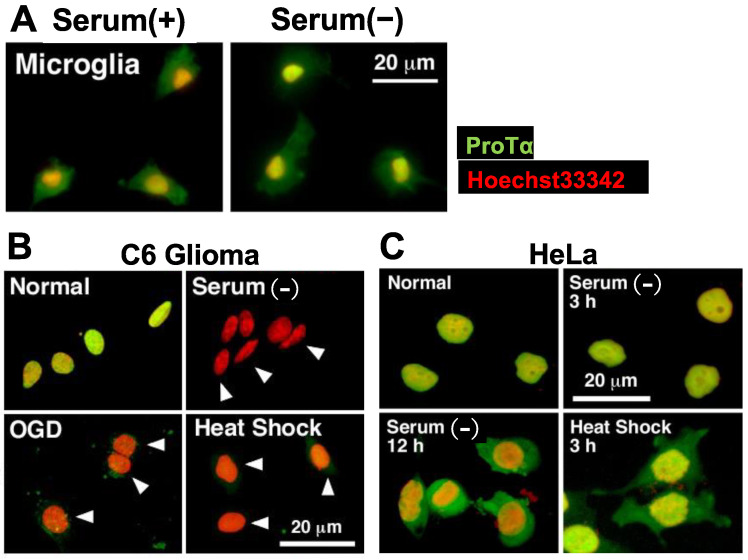
Lack of stress-induced ProTα release in microglia and HeLa cells. (**A**–**C**). Representative pictures of ProTα immunocytochemistry in rat microglia (**A**), C6 glioma (**B**) and HeLa cells (**C**) cultured with various types of stress. (**A**). Lack of ProTα release from microglia by serum deprivation stress. ProTα is detected throughout the cell both in the presence or absence of serum. (**B**). ProTα is detected in the nucleus of C6 glioma cells in the presence of serum, while no ProTα is detected in the nucleus and cytosol after the treatment with oxygen glucose deprivation (OGD, glucose-free, 1% O_2_, 3 h) or heat shock (pre-heat treatment at 42 °C for 90 min, followed by incubation at 37 °C for 3 h). (**C**). Lack of ProTα release from HeLa cells by serum deprivation or heat shock stress. ProTα is detected throughout the cell in the presence of serum deprivation or heat shock stress.

Unlike in the case of cultured neurons and astrocytes, ProTα is not localized in the nucleus of cultured microglia. In addition, ProTα release was not caused by serum deprivation stress (Figure 4A). Mechanisms underlying the lack of this release from microglia remain to be fully determined, but some of them may be explained by the fact that v-SNARE Syt-1, t-SNARE Stx-1 and ATP8A2 are substantially missing in the microglia (Supplementary Figures S1A and S2A). Analogous events were also observed in the culture of HeLa cells, which also lack Syt-1 and Stx-1. The extracellular release of ProTα from C6 glioma cells was also observed by serum deprivation, oxygen glucose deprivation (OGD) or heat shock, while no release was observed in HeLa cells (Figure 4B,C, Supplementary Figures S1B,C and S2B). In HeLa cells, ProTα was localized in the nucleus in the presence of serum, while it became distributed throughout the cell in the absence of serum, suggesting that HeLa cells have an importin-mediated ATP-dependent nuclear localization system but not a Ca^2+^-mediated extracellular flop-out system.

### 3.3. ANXA2 Flop System Underlying Anti-Stroke Actions of ProTα

**Figure 5:**
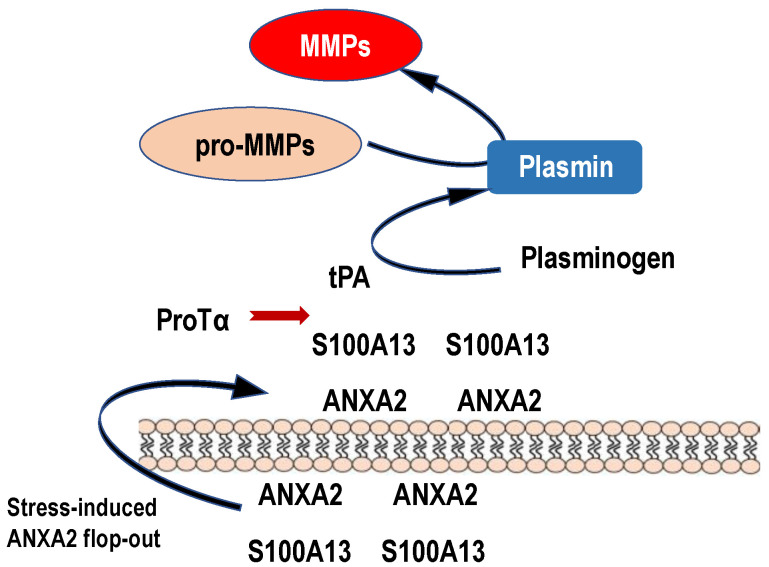
Possible roles of stress-induced ANXA2 flop-out in the plasmin and MMP production. Upon ischemic stress, protein complex comprising ProTα and S100A13 is released to the extracellular space by the force of ANXA2 flop-out mechanism. Because of high levels of Ca^2+^, S100A13 is expected to keep binding to ANXA2. On the analogy of the previously reported model, in which S100A10 bound to ANXA2 could be a receptor for tPA [60], endogenous or exogenous tPA binds to externalized S100A13 on the vascular cells or other cells in vicinity and causes the production of plasmin and MMPs. Produced MMPs may cause a hemorrhage by degrading tight junction proteins. Endogenous ProTα released upon stress may bind to S100A13 and prevent the MMP production to some extent by the inhibition of tPA binding to S100A13. Exogenous tPA may produce large amounts of MMPs via binding to S100A13 and cause hemorrhages, which are suppressed by co-administration of ProTα.

Exogenously administered ProTα shows various beneficial actions against ischemia–reperfusion stress in the cerebral artery occlusion (MCAO)-induced focal ischemia model, the bilateral common carotid artery occlusion-induced global ischemia model and the retinal ischemia–reperfusion model [27,30]. The blockade of the beneficial actions of endogenously released ProTα were also observed using neutralized anti-ProTα IgG or antisense oligodeoxynucleotide in MCAO and retinal ischemia–reperfusion models [28,29]. The most striking actions of ProTα have been recently reported [59], in which a hemorrhage induced by late treatment (6 h) with a tissue plasminogen activator (tPA) was abolished with the co-administration of ProTα. Underlying mechanisms are closely related to the inhibition of matrix metalloprotease (MMPs) production in microglia following MCAO. On the other hand, in the case of late treatment with a tPA, the MMP production was further observed in CD31-positive vascular endothelial cells, and the co-administration with ProTα showed a complete inhibition [59] (Supplementary Figure S3). Although the precise mechanisms of ProTα’s potent beneficial actions against ischemic and tPA-induced MMP production remain elusive, PS-ANXA2 flop-out may be related to them. Regarding this mechanism, there is a report that the S100A10–ANXA2 complex could be a potential tPA binding site [60]. Thus, under the MCAO condition, tPA may stimulate MMP activities and damage S100A10–ANXA2-attached brain cells, including endothelial cells as well as neurons and microglia. As ProTα is able to tightly bind to S100A13 in the presence of Ca^2+^ (extracellular concentration), exogenously administered ProTα may block the tPA binding and subsequent MMP-mediated damage. At initial or weak MCAO, released endogenous ProTα may also inhibit the binding of naturally occurring tPA (Figure 5).

## 4. Conclusions

The ANXA2 flop-out-type non-vesicular release of ProTα is a unique mechanism and, it looks distinct from known mechanisms through the membrane pores made of GSDMD or MLKL. Instead of membrane pores, the proposed mechanism includes TMEM16F scramblase, which plays a role in the process of phosphatidylserine translocation from the inner to the outer leaflet of the plasma membrane and is activated by very high Ca^2+^ concentrations [61]. In addition, ANXA2 binds membrane phospholipids in the presence of Ca^2+^ and plays a role in vesicle budding, fusion, internalization and membrane repair via lipid segregation [62]. Although we need to examine what types of DAMPs/alarmins use this mechanism, there is a similar example within the heat-shock-stress-induced release of FGF-1, which is co-released with S100A13 and Syt-1 upon serum deprivation and heat shock [34,35,63]. Interestingly, IL-1α is also co-released with S100A13 by heat shock stress, but Syt-1 is not [64]. The reason for the different involvement of Syt-1 between brain FGF-1 and IL-1α may be explained by a possibility that IL-1α release may use a different molecule instead of Syt-1 [65]. So far, three DAMPs/alarmins may use this model, although it remains elusive whether ANXA2 flop-out is involved in the release of FGF-1 and IL-1α. In addition, there are reports that IL-1α is released upon inflammasome activation and subsequent GSDMD-mediated mechanisms [66,67], suggesting that DAMPs/alarmins could be released via different mechanisms upon different types of stress and different cell types.

## Supplementary Materials

The following supporting information can be downloaded at https://www.mdpi.com/article/10.3390/cells12121569/s1, Figure S1: Cell type-specific ProTα release induced by various types of stress; Figure S2: Cell type-specific expression of key molecules involved in stress-induced ProTα release; Figure S3: Proposed schematic mechanisms underlying the blockade of hemorrhage by ProTα.
