# Supplementary material for: Non-Vesicular Release of Alarmin Prothymosin α Complex Associated with Annexin-2 Flop-Out
Source: Cells. 2023 Jun 6;12(12):1569. doi: 10.3390/cells12121569 (PMC10296757; doi:10.3390/cells12121569)
Supplement: Supplementary file 1 [file cells-12-01569-s001.zip › ProTa release_SupFig.Legends.pptx]

## Slide 1
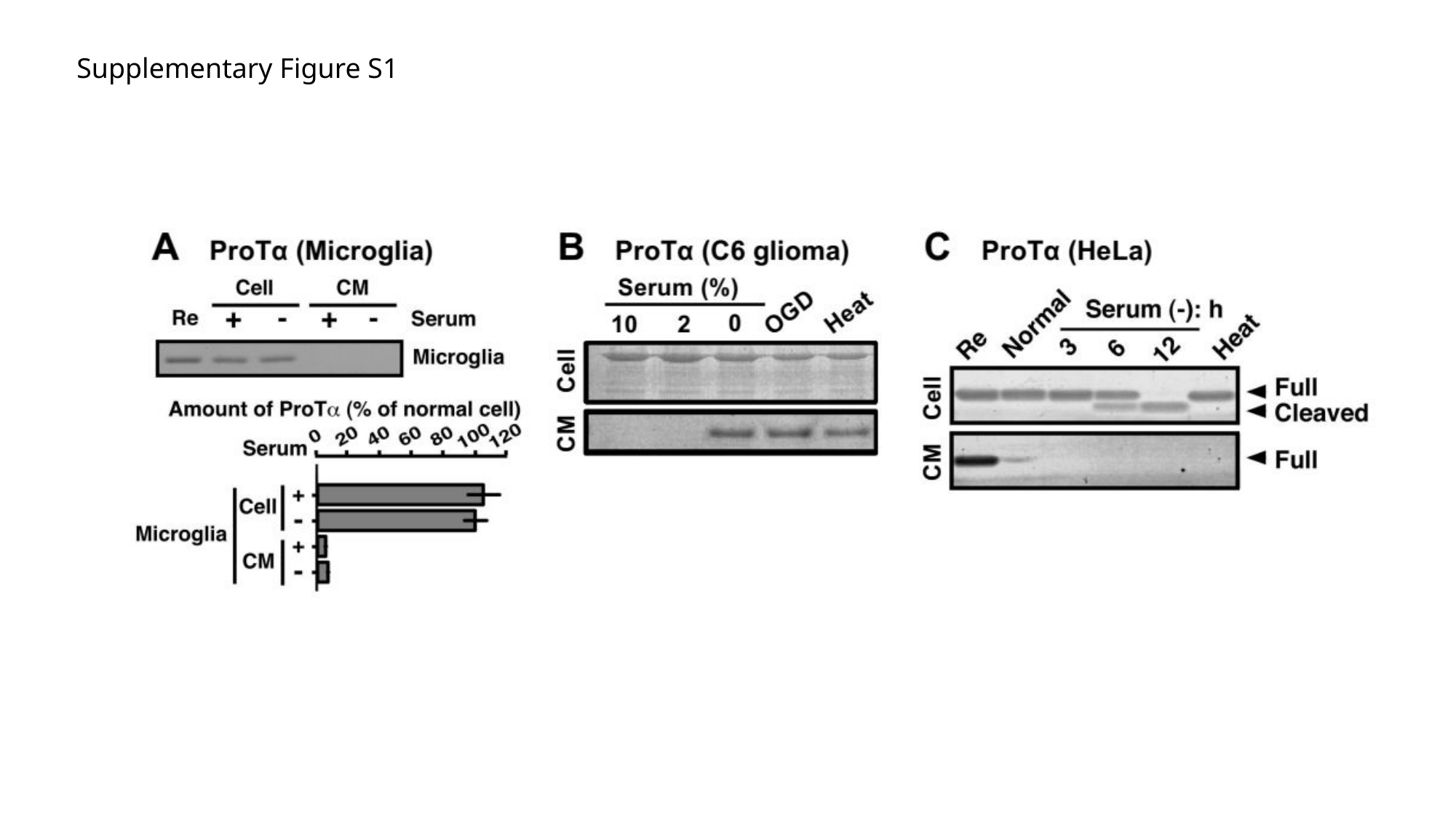

Supplementary Figure S1

## Slide 2
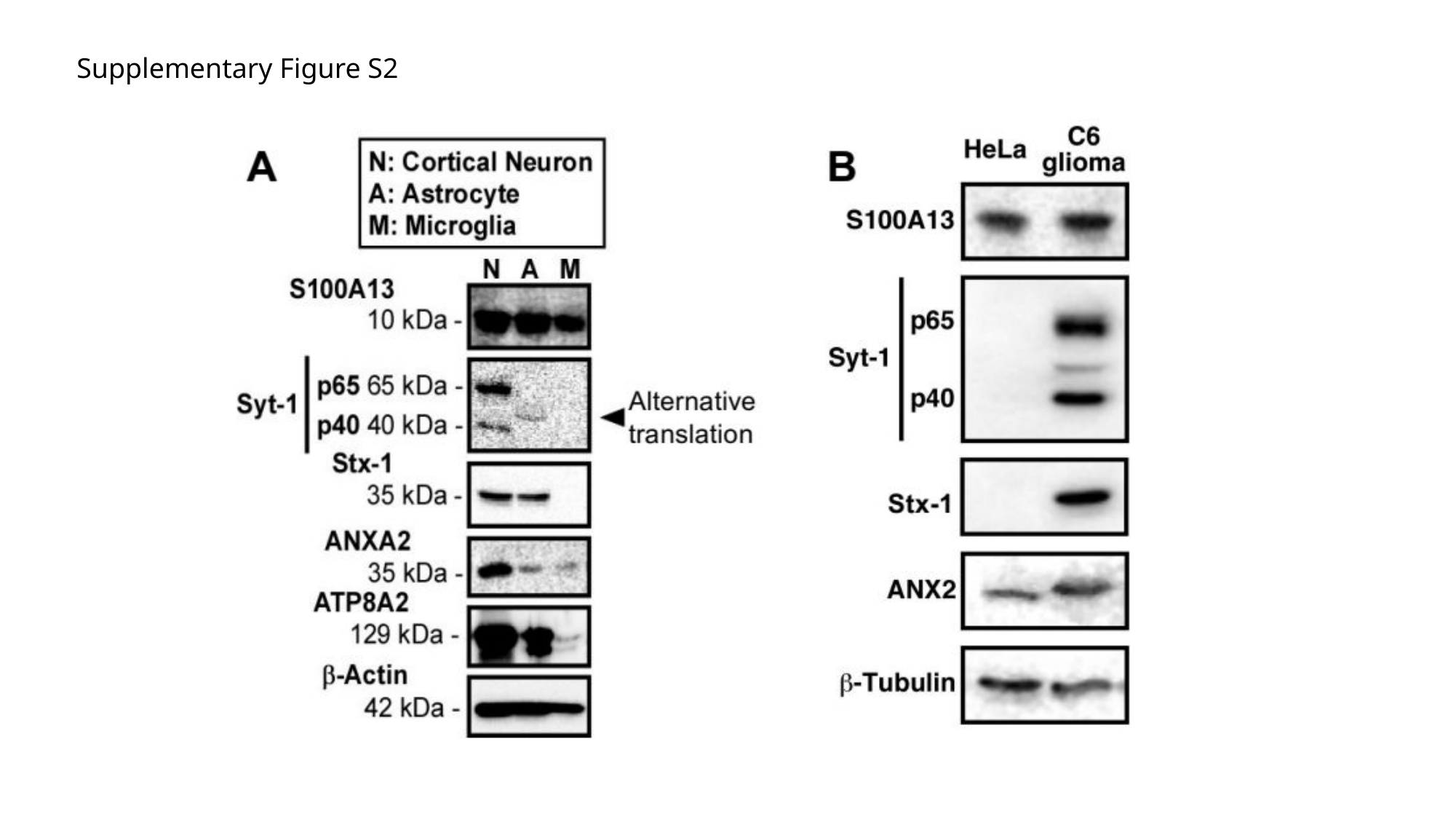

Supplementary Figure S2

## Slide 3
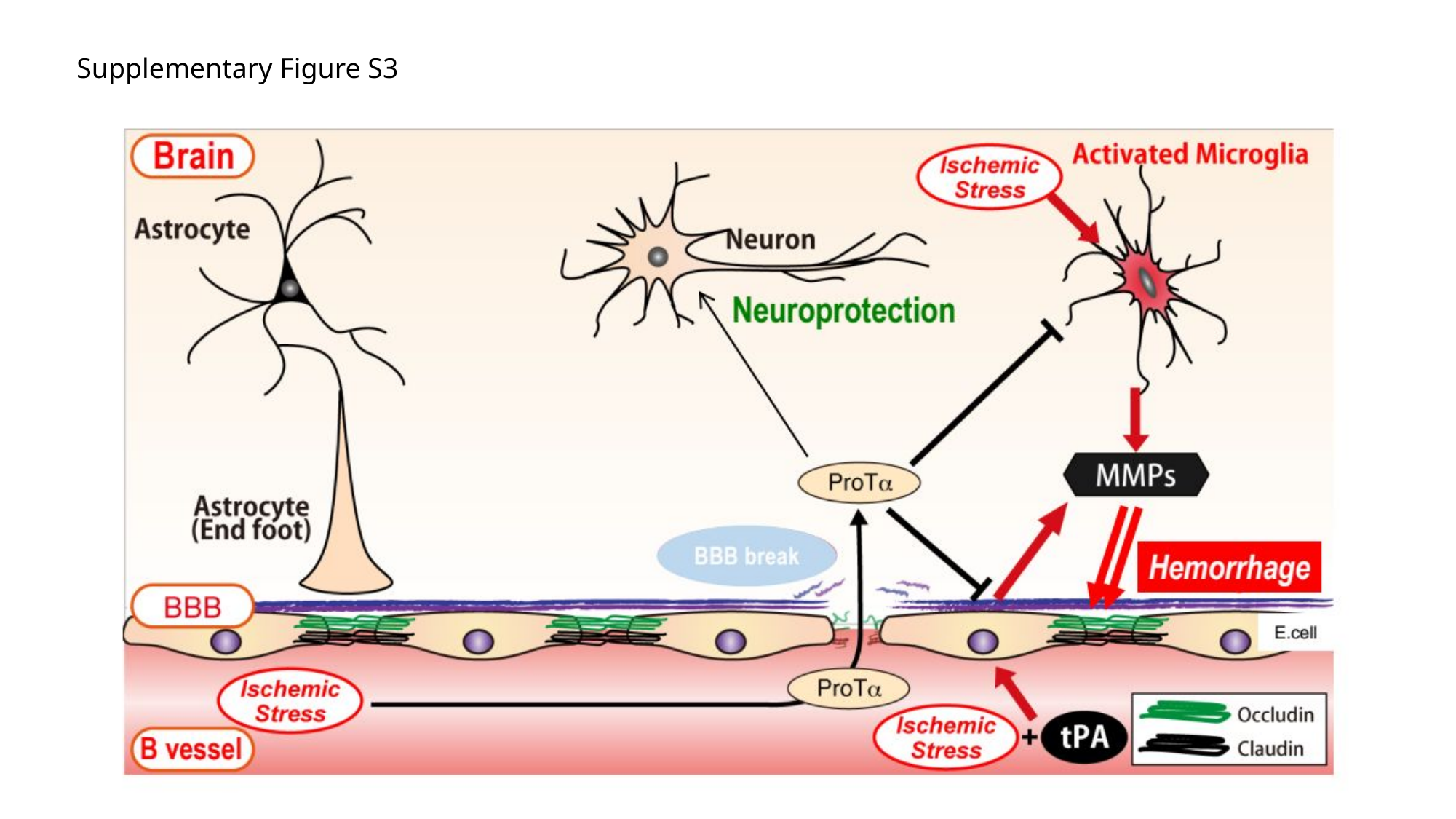

Supplementary Figure S3

## Slide 4
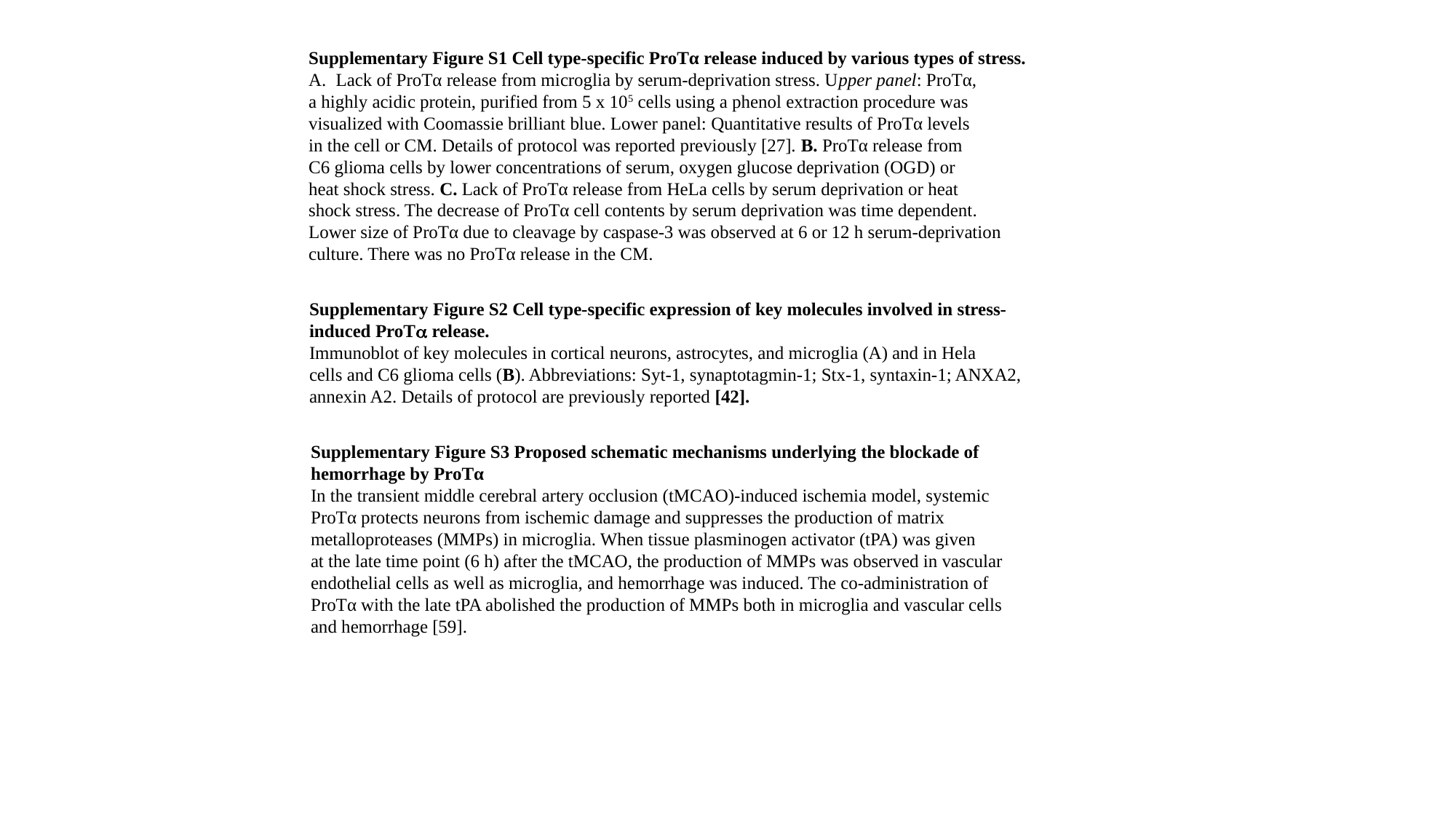

Supplementary Figure S1 Cell type-specific ProTα release induced by various types of stress.
Lack of ProTα release from microglia by serum-deprivation stress. Upper panel: ProTα,
a highly acidic protein, purified from 5 x 10^5^ cells using a phenol extraction procedure was
visualized with Coomassie brilliant blue. Lower panel: Quantitative results of ProTα levels
in the cell or CM. Details of protocol was reported previously [27]. B. ProTα release from
C6 glioma cells by lower concentrations of serum, oxygen glucose deprivation (OGD) or
heat shock stress. C. Lack of ProTα release from HeLa cells by serum deprivation or heat
shock stress. The decrease of ProTα cell contents by serum deprivation was time dependent.
Lower size of ProTα due to cleavage by caspase-3 was observed at 6 or 12 h serum-deprivation
culture. There was no ProTα release in the CM.
Supplementary Figure S2 Cell type-specific expression of key molecules involved in stress-
induced ProTα release.
Immunoblot of key molecules in cortical neurons, astrocytes, and microglia (A) and in Hela
cells and C6 glioma cells (B). Abbreviations: Syt-1, synaptotagmin-1; Stx-1, syntaxin-1; ANXA2,
annexin A2. Details of protocol are previously reported [42].
Supplementary Figure S3 Proposed schematic mechanisms underlying the blockade of
hemorrhage by ProTα
In the transient middle cerebral artery occlusion (tMCAO)-induced ischemia model, systemic
ProTα protects neurons from ischemic damage and suppresses the production of matrix
metalloproteases (MMPs) in microglia. When tissue plasminogen activator (tPA) was given
at the late time point (6 h) after the tMCAO, the production of MMPs was observed in vascular
endothelial cells as well as microglia, and hemorrhage was induced. The co-administration of
ProTα with the late tPA abolished the production of MMPs both in microglia and vascular cells
and hemorrhage [59].
